# Is acupoint injection the optimal way to administer mecobalamin for diabetic peripheral neuropathy? A meta-analysis and trial sequential analysis

**DOI:** 10.3389/fneur.2023.1186420

**Published:** 2023-10-18

**Authors:** Fei Zhang, Yunfeng Yu, Shuang Yin, Gang Hu, Xinyu Yang, Keke Tong, Rong Yu

**Affiliations:** ^1^College of Chinese Medicine, Hunan University of Chinese Medicine, Changsha, Hunan, China; ^2^The First Hospital of Hunan University of Chinese Medicine, Changsha, Hunan, China; ^3^The Hospital of Hunan University of Traditional Chinese Medicine, Changde, Hunan, China

**Keywords:** acupoint injection, mecobalamin, diabetes, peripheral neuropathy, systematic review

## Abstract

**Objective:**

Mecobalamin is a commonly used drug in the treatment of diabetic peripheral neuropathy (DPN). This study aimed to systematically evaluate the efficacy and safety of acupoint injection of mecobalamin for DPN.

**Methods:**

Relevant clinical trials on acupoint injection of mecobalamin for DPN published before 31 January 2023 were searched in eight commonly used databases. After screening and confirming the included studies, meta-analysis and trial sequential analysis were performed.

**Results:**

A total of 10 relevant studies were confirmed, and the total sample size was 927 cases. On the efficacy endpoints, meta-analysis showed that compared with other administration methods, acupoint injection of mecobalamin significantly increased the clinical effective rate by 27% [RR = 1.27, 95% CI = (1.19, 1.36), *P* < 0.00001], motor nerve conduction velocity (median nerve) by 5.93 m/s [MD = 5.93, 95% CI = (4.79, 7.07), *P* < 0.00001], motor nerve conduction velocity (common peroneal nerve) by 5.66 m/s [MD = 5.66, 95% CI = (2.89, 8.43), *P* < 0.0001], sensory nerve conduction velocity (median nerve) by 4.83 m/s [MD = 4.83, 95% CI = (3.75, 5.90), *P* < 0.00001], and sensory nerve conduction velocity (common peroneal nerve) by 3.60 m/s [MD = 3.60, 95% CI = (2.49, 4.71), *P* < 0.00001], and trial sequential analysis showed these benefits were conclusive. In terms of safety endpoints, meta-analysis indicated that the total adverse events for acupoint injection were comparable to other methods of administration, and trial sequential analysis suggested that the results needed to be validated by more studies. Subgroup analysis demonstrated that the benefits of acupoint injections of mecobalamin were not limited by the dose, duration of treatment, or number of acupoints reported in the included studies. Harbord's test showed no significant publication bias (*P* = 0.106).

**Conclusion:**

The efficacy of acupoint injection of mecobalamin for DPN was significantly better than other administrations, and its safety was comparable to other administrations. Therefore, acupoint injection may be the optimal method of mecobalamin for DPN.

**Systematic review registration:**

https://www.crd.york.ac.uk/prospero/display_record.php?RecordID=454120, identifier: CRD42023454120.

## 1. Introduction

Diabetic peripheral neuropathy (DPN) is a peripheral nerve disorder caused by persistent hyperglycemia (1), which is one of the common chronic complications of diabetes (2). DPN mainly manifests as abnormal sensation in distal limbs, symmetrical pain, and movement disorder (3). It is a major cause of disability, foot ulceration, and amputation, which seriously affects the quality of patients' lives (4). Epidemiological studies have shown that ~10% to 20% of patients are diagnosed with peripheral neuropathy at the same time as diabetes (5) and that 50% to 66% of all diabetic patients eventually develop DPN (6). Diabetes patients are reported to spend $663.2 billion per year on treatment. If they got DPN at the same time, the cost would be more than double (7). This is a huge economic burden on individuals, families, health systems, and society. Currently, the goal of treating DPN is to prevent the progression of neuropathy and neurological dysfunction (8). Clinically, nerve-nourishing drugs are often used in combination on the basis of controlling blood sugar.

Mecobalamin is a commonly used clinical drug for nerve nutrition and is essentially endogenous vitamin B12 (9). It can repair the damaged nerve tissue and relieve patients of associated symptoms by promoting the synthesis of lecithin in the medullary sinus and the metabolism of proteins, nucleic acids, and lipids (9). High doses of mecobalamin have been reported to increase the motor nerve conduction velocity of the peroneal nerve by 1.61 m/s and the sensory nerve conduction velocity of the peroneal nerve by 2.73 m/s (10). Although nutritional drugs such as mecobalamin have slowed the progression of DPN to some extent, their clinical efficacy remains limited (11). Therefore, the intervention of novel therapeutic strategies is urgently needed.

In recent years, acupoint injection has attracted more and more attention from researchers (12, 13). Acupoint injection is a way of administering drugs by injecting drugs at acupoints or tender points (14), which is convenient for operation, cost slow, and has few side effects (15). Drug acupoint injections can simultaneously exert the therapeutic effect of drugs and acupoint stimulation, which may lead to a better therapeutic effect (16). Acupoint injections of mecobalamin have been reported to be more effective in reducing clinical symptoms in patients with DPN compared to intramuscular injections, and acupoint injections may be a more effective way to administer the drug (17). However, the research on acupoint injection of mecobalamin is still at the stage of single-center randomized controlled trials, and there is no higher-quality evidence-based evidence for reference. Therefore, this study intends to use meta-analysis and trial sequential analysis (TSA) to systematically evaluate the clinical efficacy and safety of acupoint injection of mecobalamin.

## 2. Materials and methods

This study strictly followed the systematic review and meta-analysis methodology of the preferred reporting items for systematic reviews and meta-analyses (18) and was registered on PROSPERO (CRD42023454120).

### 2.1. Literature search

The literature on acupoint injection of mecobalamin for diabetic peripheral neuropathy was searched in four commonly used Chinese databases (China National Knowledge Infrastructure, Chinese Biology Medicine, VIP, and WanFang) and four general English databases (Web of Science, Embase, the Cochrane Library, and PubMed). The subject terms were mecobalamin, acupoint injection, diabetes, peripheral neuropathy, etc. The free terms were supplemented by the Mesh and the Cochrane Library databases, after which the free terms were combined with the subject terms to build a complete search formula. The search is capped at January 2023, with no language or region restrictions.

### 2.2. Inclusion and exclusion criteria

The inclusion criteria were as follows: (i) The clinical trials with randomized controlled design. (ii) The patients met the diagnostic criteria for type 2 diabetes established by the World Health Organization in 1999 and had peripheral neuropathy (19–25): (1) abnormal sensation in the limbs; (2) markedly diminished or absent superficial and deep reflexes; (3) electrophysiological examination suggestive of a slowing down of the nerve conduction velocities; (4) exclusion of other causes of neuropathy. (iii) The administration of mecobalamin to the DPN patients in the experimental group was by acupoint injection, while the administration of mecobalamin to the control group was by other methods (oral, intramuscular, and intravenous injection). (iv) The primary efficacy endpoint was the clinical effective rate. The clinical effective rate was the effective number divided by the total number. Effective was defined as a reduction in clinical symptoms and an improvement in achilles and knee reflexes. Secondary efficacy endpoints included motor nerve conduction velocity and sensory nerve conduction velocity. The safety endpoint was total adverse events.

The exclusion criteria were as follows: (i) The same research findings had been repeatedly reported. (ii) The data was not available. (iii) The included objects covered special groups such as children.

### 2.3. Literature screening, statistics, and quality evaluation

After importing all the literature to be screened into NoteExpress and excluding duplicates, the target literature was gradually screened according to the inclusion criteria. After identifying the included literature, basic information on the clinical trials was extracted and a table of basic characteristics was drawn up. Afterward, the risk of bias in clinical trials was evaluated in accordance with Cochrane guidelines. These studies were completed independently by FZ and YY. SY was in charge of making decisions when there were disagreements.

### 2.4. Statistical analysis

Revman 5.3 software and TSA0.9.5.10 Beta were used to perform meta-analysis and trial sequential analysis, respectively. When the indicator was a continuous variable, the mean difference (MD) was used as the effect size. When the indicator was a dichotomous variable, the relative risk (RR) was used as the effect size. The heterogeneity of each indicator was evaluated by the *I*^2^ value, and the corresponding effect model was selected according to the *I*^2^ value. When *I*^2^ < 50%, a fixed effects model was chosen to analyze the data. When *I*^2^ ≥ 50%, a random effects model was selected to analyze the data. In the trial sequential analysis, the indicator was indicated to be conclusive when the Z curve reached a boundary value. Harbord-weighted linear regression was employed to test for publication bias, with no significant publication bias if *P*-value > 0.1. The quality evaluation of evidence drew on the GRADE guidelines to comprehensively evaluate the quality of evidence for each indicator.

## 3. Results

### 3.1. Literature search results

**Figure 1:**
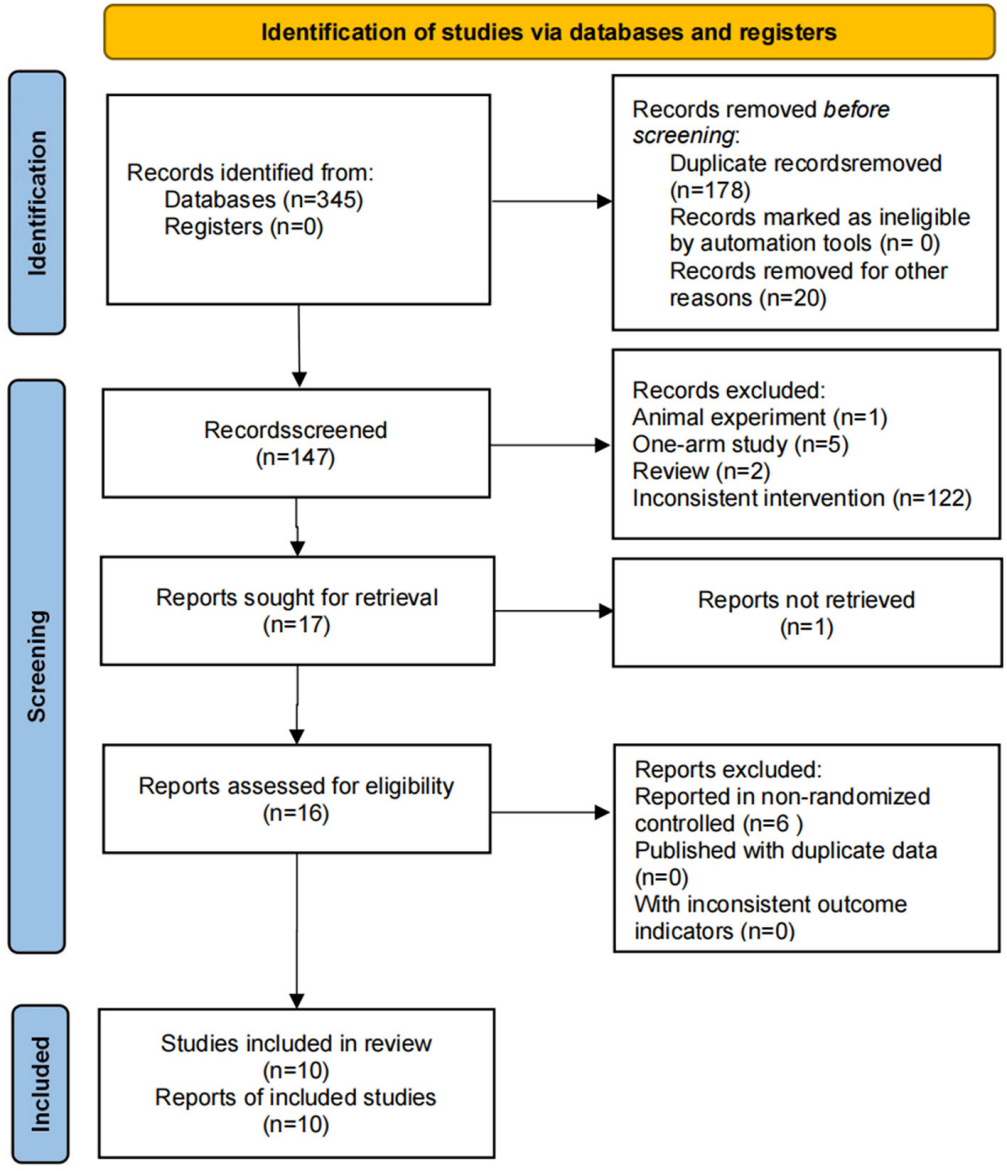
Flow chart of literature screening.

A total of 345 relevant publications were obtained from the screening. Among them, 198 studies were eliminated due to duplication and other reasons, 130 studies were excluded due to inconsistent topics (1 animal experiment, 5 single-arm studies, 2 reviews, and 122 studies with incompatible intervention programs), 1 literature was removed because no report was retrieved, and 6 studies were eliminated because of non-randomized controlled trials. Finally, 10 clinical studies (26–35) were included, as shown in Figure 1.

### 3.2. Basic characteristics of the included studies

A total of 10 clinical studies (26–35) published between 2009 and 2022 were included, and the research centers were all in China. The total sample size was 927 cases, including 464 cases in the experimental group and 463 cases in the control group. The basic characteristics are shown in Table 1. The position, regional anatomy, and innervation of the acupoints are shown in Table 2.

**Table 1:** Table of basic characteristics of the included studies.

**References**	**Number randomized (E/C)**	**Diagnostic criteria**	**Male (%)**	**Age (years)**	**Disease duration (years)**	**Acupoint**	**Intervention**	**Comparison**	**Treatment duration (day)**
Cao (26)	78/78	Other	51.3	54.0	/	ST36	Mecobalamin ia 0.5 mg qd	Mecobalamin iv 0.5 mg qd	15
Guo et al. (27)	30/30	(19)	45.0	56.8	4.6	ST36	Mecobalamin ia 0.5 mg qd	Mecobalamin im 0.5 mg qd	28
Liu (28)	50/50	Other	66.0	56.7	/	ST36	Mecobalamin ia 0.5 mg qd	Mecobalamin im 0.5 mg qd	14
Mo et al. (29)	40/42	(20)	53.7	59.0	/	ST36, SP6	Mecobalamin ia 0.5 mg qd	Mecobalamin im 0.5 mg qd	15
Sun and Han (30)	40/40	Other	/	/	/	ST36, SP6	Mecobalamin ia 0.5 mg qd	Mecobalamin im 0.5 mg qd	30
Wen (31)	35/35	(21)	51.4	54.8	8.1	LI4, LI11, ST36, SP6, GB34	Mecobalamin ia 1.25 mg q2d	Mecobalamin po 0.5 mg tid	30
Xie et al. (32)	30/30	(22)	63.3	56.6	5.7	SP10	Mecobalamin ia 0.5 mg q2d	Mecobalamin po 0.5 mg tid	30
Xie et al. (33)	25/25	(23)	48.3	59.4	7.6	SP10, ST36	Mecobalamin ia 0.5 mg q2d	Mecobalamin im 0.5 mg q2d	56
Yang et al. (34)	38/35	(24)	58.9	60.50	/	ST36, SP6, GB34, BL57	Mecobalamin ia 0.5 mg q2d	Mecobalamin iv 0.5 mg q2d	30
Zhao et al. (35)	93/93	(25)	/	/	/	ST36, SP6, GB34, BL60	Mecobalamin ia 0.5 mg qd	Mecobalamin iv 0.5 mg qd	15

**Table 2:** Position, regional anatomy, and innervation of acupoints.

**Acupoint**	**Position**	**Regional anatomy**
ST36	On the lateral side of the lower leg, 3 cun below the Dubi (ST35), on the line between the Dubi (ST35) and Jiexi (ST41)	Skin, subcutaneous tissue, tibialis anterior muscle, deep peroneal nerve and anterior tibial artery, interosseous membrane of the lower leg, tibialis posterior muscle
SP6	On the medial side of the lower leg, 3 cun above the tip of the inner ankle, posterior to the medial border of the tibia	Skin, subcutaneous tissue, flexor digitorum longus, tibialis posterior, lesser flexor digitorum longus
LI4	On the back of the hand, the midpoint of the radial side of the second metacarpal	Skin, subcutaneous tissue, first interosseous dorsalis muscle, bunion muscle
LI11	In the elbow region, with the elbow flexed, the midpoint of the line between the lateral epicondyle of the humerus and the radial end of the transverse elbow stripe	Skin, subcutaneous tissue, radial long and short extensor carpi radialis longus, brachioradialis muscle, radial nerve and anterior branch of radial parasympathetic artery, brachioradialis muscle
GB34	On the lateral side of the lower leg, in the depression below the anterior aspect of the head of the fibula	Skin, subcutaneous tissue, peroneus longus, common peroneal nerve, extensor digitorum longus muscle
SP10	In the anterior femoral region, 2 cun above the medial end of the patellar base, when the medial femoral muscle is elevated	Skin, subcutaneous tissue, medial femoral muscle
BL57	In the posterior region of the lower leg, at the angle where the gastrocnemius muscle belly meets the tendon	Skin, subcutaneous tissue, gastrocnemius muscle, flounder muscle, tibial nerve, posterior tibial artery and vein
BL60	On the lateral side of the ankle, in the depression between the tip of the lateral ankle and the Achilles tendon	Skin, subcutaneous tissue, loose connective tissue

### 3.3. Risk of bias assessment

**Figure 2:**
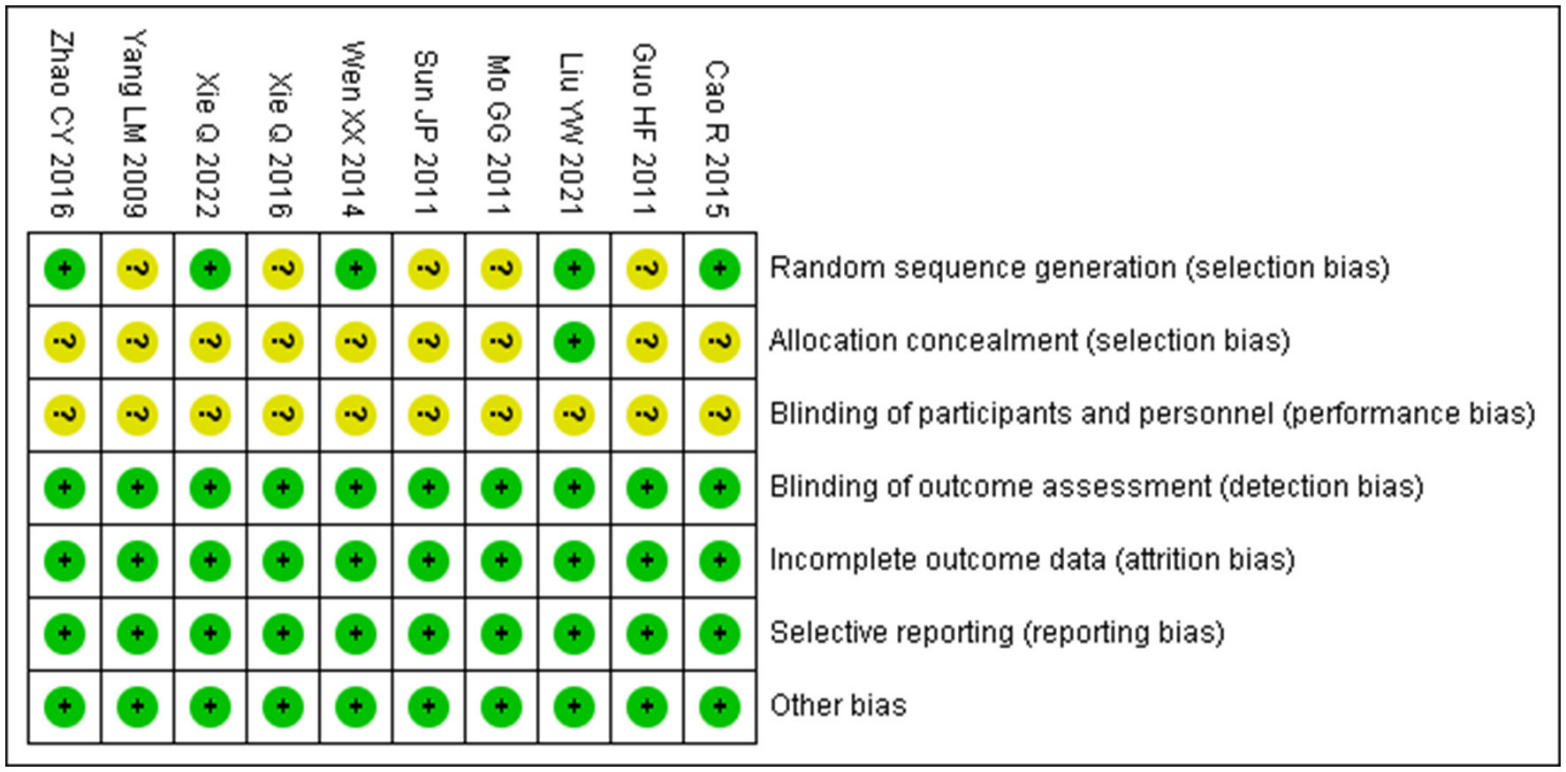
Risk of bias graph.

The risk of bias assessment showed that the risk of bias was unclear for the randomized design in five studies, allocation concealment in nine studies, and blinding of participants to the intervention in 10 studies, with the remaining areas at low risk of bias, as shown in Figure 2.

### 3.4. Meta-analysis results

#### 3.4.1. Efficacy endpoints

##### 3.4.1.1. Primary efficacy endpoint

**Figure 3:**
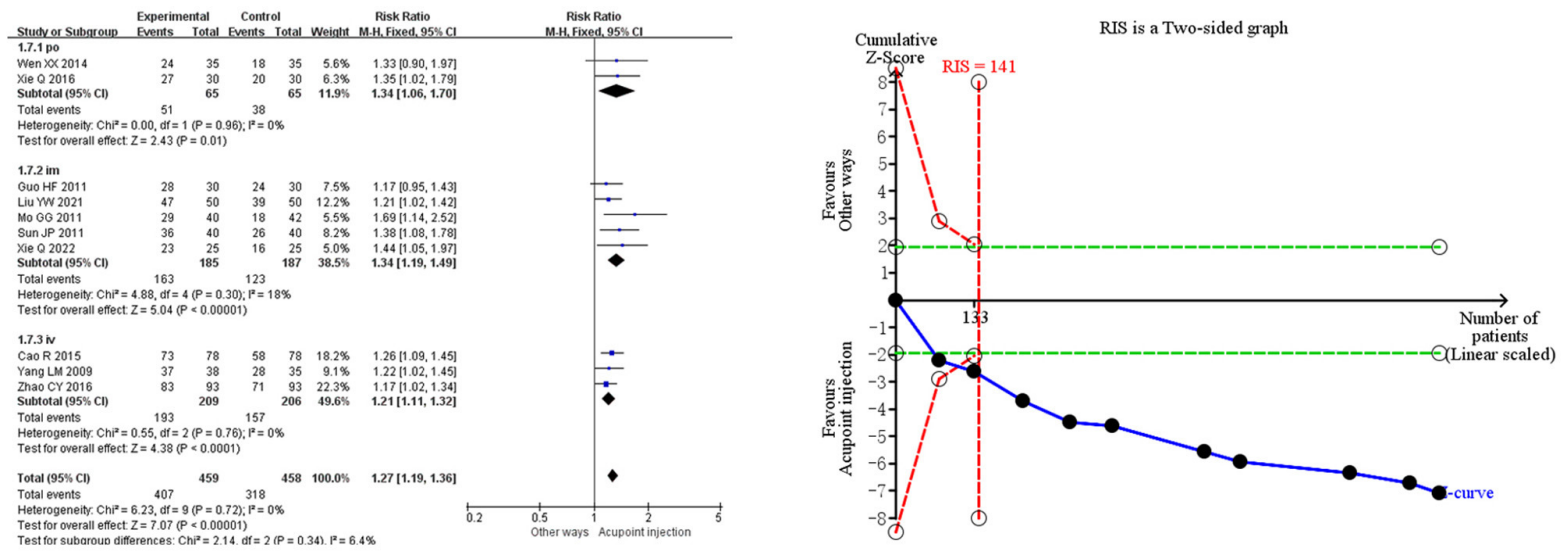
Meta-analysis and trial sequential analysis of the primary efficacy endpoints for acupoint injections of mecobalamin for diabetic peripheral neuropathy.

Meta-analysis demonstrated that acupoint injection of mecobalamin significantly increased the clinical effective rate [RR = 1.27, 95% CI (1.19, 1.36), *P* < 0.00001] by 27% compared to other delivery methods. Sensitivity analyses showed low sensitivity for the clinical effective rate combined analyses, suggesting that the results were robust. In the subgroup analysis, acupoint injection of mecobalamin increased the clinical effective rate by approximately 34% relative to oral administration [RR = 1.34, 95% CI (1.06, 1.70), *P* = 0.01], approximately 34% relative to intramuscular injection [RR = 1.34, 95% CI (1.19, 1.49), *P* < 0.00001], and ~21% relative to intravenous injection [RR = 1.21, 95% CI (1.11, 1.32), *P* < 0.0001]. Trial sequential analysis indicated that the cumulative Z-value for the clinical effective rate crossed the TSA boundary in the second study, suggesting that the results observed in the current information set were conclusive. The quality assessment of the evidence showed that the quality of evidence for the clinical effectiveness rate was moderate, as shown in Figure 3.

##### 3.4.1.2. Secondary efficacy endpoints

**Figure 4:**
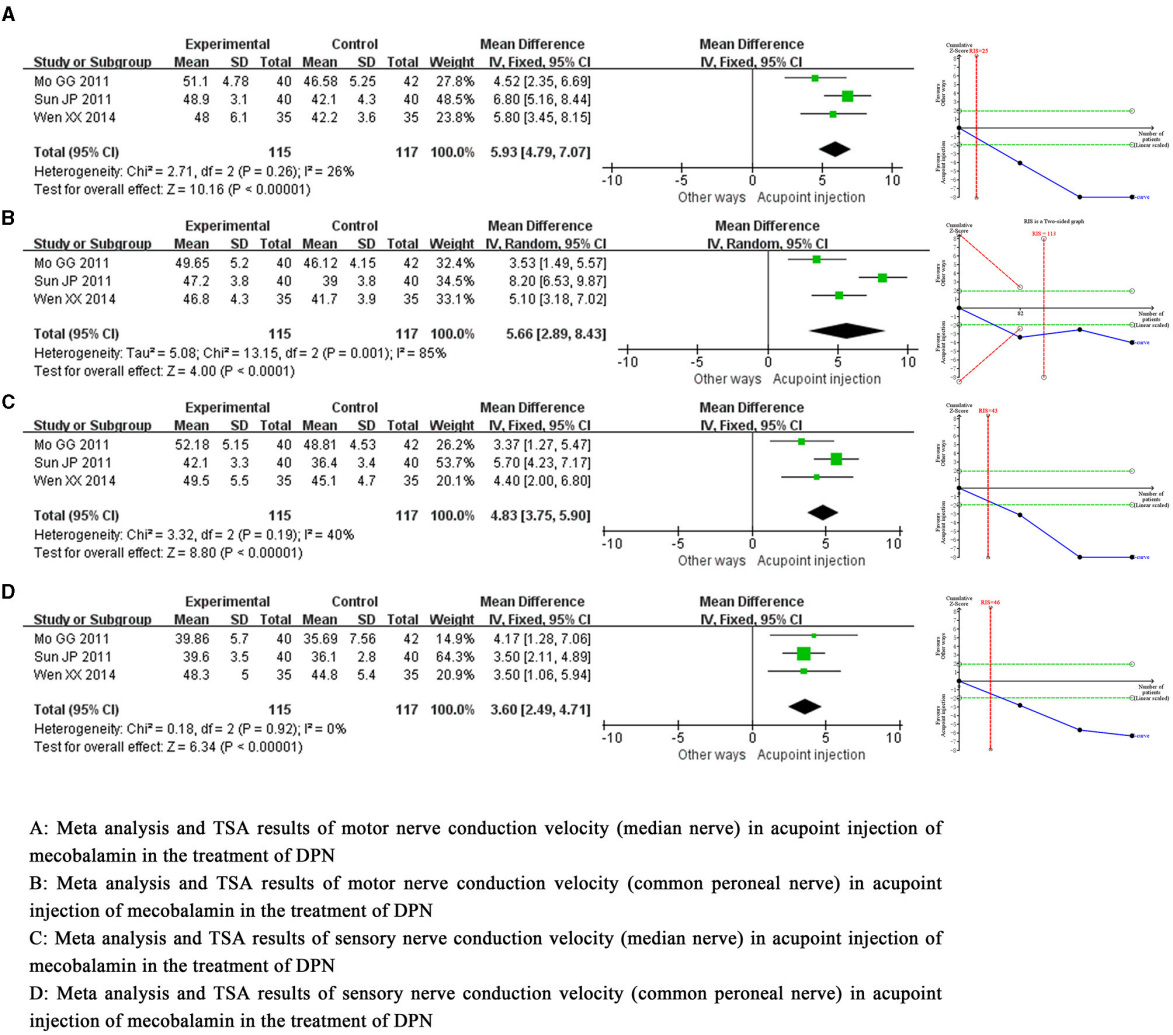
Meta-analysis and trial sequential analysis of secondary efficacy endpoints for acupoint injection of mecobalamin for diabetic peripheral neuropathy.

Meta-analysis demonstrated that acupoint injection of mecobalamin significantly increased not only motor nerve conduction velocity (median nerve) by 5.93 m/s [MD = 5.93, 95% CI = (4.79, 7.07), *P* < 0.00001] and motor nerve conduction velocity (common peroneal nerve) by 5.66 m/s [MD = 5.66, 95% CI = (2.89, 8.43), *P* < 0.0001] but also sensory nerve conduction velocity (median nerve) by 4.83 m/s [MD = 4.83, 95% CI = (3.75, 5.90), *P* < 0.00001] and sensory nerve conduction velocity (common peroneal nerve) by 3.60 m/s [MD = 3.60, 95% CI = (2.49, 4.71), *P* < 0.00001] compared to other delivery methods. Sensitivity analysis showed a low sensitivity and high confidence in the results for the combined motor nerve conduction velocity (common peroneal nerve). Trial sequential analysis suggested that these benefits observed for the current information set were conclusive. The quality of evidence assessment showed that the quality of evidence for motor nerve conduction velocity (common peroneal nerve) was low, and the quality of evidence for other indicators was moderate, as shown in Figure 4.

#### 3.4.2. Safety endpoints

**Figure 5:**
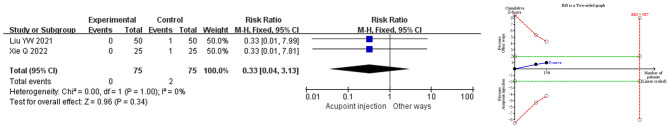
Meta-analysis and trial sequential analysis of safety endpoints for acupoint injection of mecobalamin for diabetic peripheral neuropathy.

Meta-analysis demonstrated that the total adverse events for acupoint injection of mecobalamin were comparable to other delivery methods. Trial sequential analysis indicated that the cumulative Z-value for total adverse events did not yet meet the desired information set, suggesting that the results need to be validated in more relevant studies. The quality assessment of evidence showed that the quality of evidence for total adverse events was low, as shown in Figure 5.

### 3.5. Subgroup analysis

Subgroups of “0.5 mg q2d”, “0.5 mg qd,” and “1.25 mg q2d” were set up using the dose of acupoint injection as the category. The results demonstrated that acupoint injection of mecobalamin at “0.5 mg q2d” and “0.5 mg qd” significantly improved the clinical efficiency, while the clinical efficacy rate of “1.25 mg q2d” was comparable to that of the control group, as shown in Table 3.

**Table 3:** Subgroup analysis of acupoint injection of mecobalamin for DNP.

**Topic**	**Subgroup**	**Experimental group (events/total)**	**Control group (events/total)**	***I* ^2^**	**RR (95% CI)**	***P***
Dose	0.5 mg q2d	87/93	64/90	0	1.31 (1.14, 1.51)	0.0002
	0.5 mg qd	296/331	236/333	0	1.26 (1.17, 1.36)	< 0.00001
	1.25 mg q2d	24/35	18/35	0	1.33 (0.90, 1.97)	0.15
Course	2 weeks	269/299	214/298	14	1.25 (1.15, 1.37)	< 0.00001
	4 weeks	152/173	116/170	0	1.28 (1.15, 1.44)	< 0.0001
	6 weeks	23/25	16/25	0	1.44 (1.05, 1.97)	0.02
Number of acupoints	Single acupoint	175/188	141/188	0	1.24 (1.13, 1.36)	< 0.00001
	Multiple acupoints	232/271	177/270	0	1.30 (1.18, 1.43)	< 0.00001

RR, Risk Ratio; the clinical effective rate of the experimental group/the clinical effective rate of the control group, which is operated by Revman 5.3.

There are subgroups of “2 weeks,” “4 weeks,” and “6 weeks” based on the duration of the acupoint injection. The results indicated that acupoint injections of mecobalamin at “2 weeks,” “4 weeks,” and “6 weeks” significantly increased the clinical effective rates, as shown in Table 3.

“Single-acupoint” and “multiple-acupoints” subgroups were set by the number of acupoints injected. The results suggested that acupoint injection of mecobalamin at both “single” and “multiple” acupoints significantly improved the clinical effective rates, as shown in Table 3.

### 3.6. Assessment of publication bias

**Figure 6:**
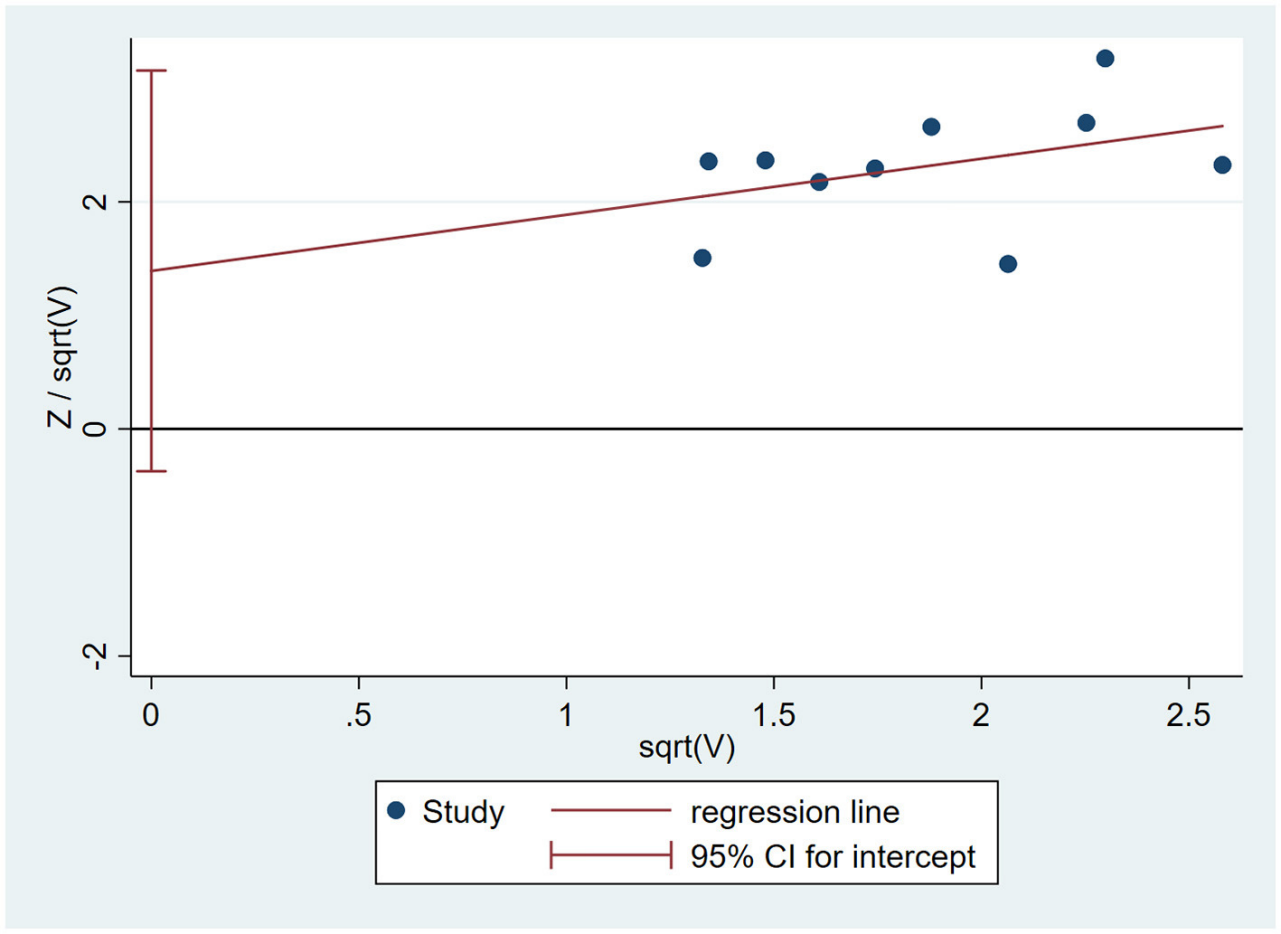
Harbord-weighted linear regression of the clinical effective rate.

Taking the clinical effective rate as the standard, the Harbord-weighted linear regression based on event rates in the experimental and control groups showed no significant publication bias in the study (*P* = 0.106), as shown in Figure 6.

## 4. Discussion

Currently, lowering blood sugar and nourishing the nerves are the main means of treating DPN (36). Although drugs such as mecobalamin and alpha-lipoic acid can delay the progression of DPN to some extent, their clinical efficacy is still limited (37). Acupoint injection is a characteristic therapy in which drugs are injected into corresponding acupoints for therapeutic purposes under the guidance of the meridian theory of Chinese medicine and is widely used in China because of its simple operation and considerable efficacy (38). In 2006, Chinese researchers first reported the application of acupoint injection of mecobalamin in patients with DPN, and they found that acupoint injection of mecobalamin was significantly better than intramuscular injection in reducing symptoms and signs (17). Subsequently, a randomized controlled trial published in 2009 confirmed that the clinical efficacy of acupoint injections of mecobalamin was significantly higher than that of intravenous injections (34). Since then, there has been increasing evidence that acupoint injections of mecobalamin may be a more effective strategy for treating DPN (39). A total of 10 clinical trials and a sample size of 927 were subjected to this meta-analysis and TSA, which aimed to comprehensively assess the benefit of acupoint injection of mecobalamin in DPN.

Meta-analysis of the primary efficacy endpoint showed that acupoint injection of mecobalamin significantly increased the clinical effective rate by 27% relative to other delivery methods, meaning that acupoint injection was significantly more effective than any oral, intramuscular, or intravenous administration. TSA demonstrated that the observed results of the current amount of information were conclusive, implying that acupoint injection of mecobalamin was superior to other delivery methods in reducing clinical symptoms and neurological signs in patients with DPN. However, it should be noted that in the subgroup analysis of the mode of administration, the oral subgroup included only two studies and a sample size of 130. This may lead to a decrease in the precision of the results, and therefore, the results remain to be validated by more studies. Compared to other delivery methods for secondary efficacy endpoints, acupoint injection of mecobalamin significantly increased the motor nerve conduction velocities by 5.93 m/s (median nerve) and 5.66 m/s (common peroneal nerve) and the sensory nerve conduction velocities by 4.83 m/s (median nerve) and 3.60 m/s (common peroneal nerve). TSA confirmed that these benefits were conclusive, suggesting that acupoint injection can effectively improve the function of the sensory nerve and motor nerve in patients with DPN. Interestingly, Sun et al. (30) found a negative correlation between the efficacy of acupoint injections of mecobalamin and the duration of DPN disease, implying that the earlier the use of acupoint injections of mecobalamin the greater the benefit may be. In addition, some of the included studies (32, 33, 35) also reported the benefits of acupoint injection of mecobalamin in Substance P (SP) and the vibration perception threshold test (VPT). SP is a conductive substance of sensory neurons and is a potential biomarker of DPN (40, 41). Studies by Xie et al. (32, 33) have shown that compared with oral or intramuscular injection, acupoint injection of mecobalamin can significantly increase the serum SP level in patients with DPN, thereby improving neurological function. The VPT is the most sensitive test for detecting DPN and is also a predictor of foot injury (42, 43). Zhao et al. (35) found that acupoint injection of mecobalamin can significantly reduce VPT, thereby reducing the clinical symptoms of patients and the risk of foot ulcers. These evidences suggest that acupoint injection of mecobalamin may be a practical and effective way of drug delivery.

Subgroup analysis indicated that in the dose subgroup, the acupoint injection of mecobalamin at 0.5 mg q2d and 0.5 mg qd could significantly improve the clinical effective rate, while the clinical effective rate of 1.25 mg q2d was comparable to that of the control group. However, we cannot yet assume that acupoint injection of mecobalamin at 1.25 mg q2d is not clinically relevant as the original study also reported a benefit of 1.25 mg q2d in improving motor and sensory nerve conduction velocities (31). Therefore, we preliminarily judged that the acupoint injection of three dosages of mecobalamin can achieve benefits in patients with DPN. Acupoint injections of mecobalamin for 2, 4, and 6 weeks significantly increased the clinical effective rate in the course of the treatment subgroup, suggesting that additional benefits can be realized from acupoint injections in both short- and long-term treatment. In the number of acupoints subgroup, both single-acupoint injections and multiple-acupoint injections significantly increased the clinical effective rate, suggesting that both single-acupoint and multiple-acupoint injections improved clinical efficacy. In traditional Chinese medicine theory, ST36 has the function of regulating the spleen, stomach, qi and blood, and it is the most important acupoint for strong healthcare. Relevant studies have shown that stimulating ST36 can promote local blood circulation in the lower limbs and regulate local nerve function (44), so ST36 has been used by researchers as a key acupoint for the treatment of DPN by acupoint injection (45). In addition, SP6 is an intersection acupoint of the three yin channels of the foot, which has the effect of tonifying the liver and kidneys and regulating qi and blood. LI4 and LI11 are acupoints of the large intestine channel of the hand Yang Ming, which have the effect of regulating qi and blood and moistening the meridians. GB34 is an acupoint of the kidney channel of the foot Shao Yin, which is mainly used for treating musculoskeletal disorders. SP10 is an acupoint of the spleen channel of the foot Tai Yin, which is effective in activating and nourishing. BL57 and BL60 are located on the posterior side of the calf and lateral side of the ankle, respectively, which can dredge qi and blood in the lower limbs. All of these points have the effect of promoting the circulation of qi and blood, and stimulating them can promote blood circulation and improve nerve function (46). In summary, it can be noted that the additional benefit of acupoint injection of mecobalamin was not limited by the dose, duration of treatment, and number of acupoints reported in the included studies.

In terms of safety endpoints, meta-analysis demonstrated that the total adverse events for acupoint injection of mecobalamin were comparable to other delivery methods, suggesting that acupoint injection of mecobalamin has a good safety profile. TSA indicated that the cumulative Z-value for total adverse events had not yet reached the required information set, suggesting that the results have yet to be validated by more relevant studies. Although research results show that acupoint injection has good safety, we still need to pay attention to possible adverse events such as hematoma, nerve injury, pain, acupuncture dizziness, infection, and allergic reaction (47–49). (1) Hematoma: It is mainly caused by subcutaneous bleeding due to mistakenly puncturing a blood vessel or damaging the surrounding tissues during the operation. A small amount of bleeding can be absorbed by itself. If too much subcutaneous bleeding causes severe pain, cold compresses should be used to stop the bleeding first, and after 24 h, hot compresses should be applied to promote the absorption of congestion. During the operation, it is necessary to avoid blood vessels when inserting the needle, pump back the needle after insertion to prevent puncture of blood vessels, and immediately press the skin when the needle is pulled back so as to reduce the risk of hematoma. (2) Nerve damage: It refers to an injury of the nerve trunk during the operation that produces a strong feeling of electric shock and numbness. The nerve trunk should be avoided during the operation, and needle insertion should be done by pricking through the skin first and slowly. When the feeling of electric shock occurs, the needle should be pulled out immediately, and the direction of needle advancement should be changed. (3) Pain: In general, the pain when inserting the needle is mild, and the severe pain is mostly due to the formation of hematoma. So, avoiding the formation of hematoma can effectively reduce the occurrence of pain. The subgroup analysis of this study found that both single-point injection and multi-point injection can significantly improve clinical efficacy, so we recommend the use of ST36 single-point injection to reduce the painful stimulation of the patient during the entry of the needle, improving the patient's compliance. (4) Acupuncture dizziness: It refers to dizziness, nausea, palpitation, and sweating during the operation, mostly due to nervousness, hunger, and other factors. When this occurs, the injection should be stopped immediately, and the patient should recover before operating slowly. (5) Infection: This is mostly due to improper disinfection during the operation, and strict implementation of a standardized disinfection protocol can significantly reduce the risk of infection. (6) Allergic reaction: A small number of patients may develop symptoms such as skin rash, itching, palpitation, and asthma due to drug allergy, and the injection should be stopped immediately. In mild cases, it may disappear on its own after stopping the injection, while in severe cases, rescue is required. In order to avoid such dangerous situations, it is essential to ask for a detailed history of allergy and to perform a skin test before the operation.

In fact, acupoint injection is a special kind of intramuscular injection, which is required to perform intramuscular injection at the location of characteristic acupoints so that patients can obtain the therapeutic effect of drugs and acupoints at the same time. The additional benefit of acupoint injection compared with intramuscular injection is mainly due to the therapeutic effect induced by acupoints (50). However, the specific mechanism of this effect is still unclear. Some scholars believe that it may be achieved by stimulating the local innervation and blood circulation of acupoints to affect the nerve-endocrine-immune system (51). Therefore, theoretically, the safety of acupoint injection is equivalent to that of intramuscular injection, and they are both safe and reliable methods of administering mecobalamin. Notably, acupoint injection requires the same consumables as intramuscular injection, which means that it is a delivery method that can be used to achieve efficacy benefits without increasing the financial burden. Therefore, we believe that it may be of great clinical value in the future.

Although this study followed the guidelines closely, there are still some limitations. First of all, there is a certain amount of methodological heterogeneity. None of the studies mentioned blinding, which may increase the risk of implementation bias and reduce the confidence of the results. Second, the study included a small number of studies and a small sample size, and the quality of the evidence was low, which may have reduced the precision of the results. Third, the diagnostic criteria included in the study were related to the Diabetic Peripheral Nerve Diagnostic Code in 2009, the Chinese Guidelines for the Prevention and Treatment of Type 2 Diabetes (2017), the Chinese Guidelines for the Prevention and Treatment of Type 2 Diabetes (2013), the Practical Endocrinology in 2004, and the Chinese and Western Medicine Diagnosis and Treatment of Diabetes and its Complications in 1997. There are certain differences in the basic diagnosis of each standard, which may affect the meta-analysis. Fourth, acupoint injection is a common external treatment method of Chinese medicine, which is rarely used in countries other than China. This has resulted in the existing clinical trials being conducted in China and with Chinese subjects. This means that the results of the study may only apply to people of Chinese descent, and it is unclear how acupoint injections of mecobalamin work in other ethnic groups. Fifth, although this study clarifies the benefits of acupoint injection of mecobalamin, there is no direct comparison between different doses, courses of treatment, and the number of acupoints of acupoint injection of mecobalamin. Therefore, the optimal dosage and course of treatment of mecobalamin for acupoint injection are not yet clear.

Given the limitations of the existing research, we look forward to continuous improvement in future studies. First, on the basis of controlling relevant variables, high-quality randomized controlled double-blind trials can be continued. This can explore in depth the specific benefits of acupoint injections of mecobalamin at different doses, duration of treatment and number of acupoints, thereby exploring the optimal dosage and duration of acupoint injections of mecobalamin. Second, evaluation indicators such as SP and VPT can be added to study in depth the underlying mechanisms of acupoint injection of mecobalamin in treating DPN. Third, a research center could be established in European, American, and African countries to further explore the effects of acupoint injections of mecobalamin in people of European, American, and African descent. We hope that more scholars will focus on acupoint injection of mecobalamin for treating DPN in the future and that it will bring new hope to patients with DPN.

## 5. Conclusion

The efficacy of acupoint injections of mecobalamin for DPN was significantly better than other administrations, and its safety profile was comparable. Acupoint injections may be the best way to administer mecobalamin for DPN.

## Data availability statement

The original contributions presented in the study are included in the article/supplementary material, further inquiries can be directed to the corresponding author.

## Author contributions

FZ conceived the article and co-authored the first draft with YY. FZ, YY, and SY were responsible for literature and data. GH, KT, and XY were responsible for the methods and analysis. RY supervised the review and revised the manuscript. All authors contributed to the article and approved the submitted version.
